# On the Resorption of Pleuritic Exudations

**Published:** 1861-09

**Authors:** 


### On the Resorption of Pleuritic Exudations.

BY PROF. SKODA.

The resorption of pleuritic exudations frequently takes place very slowly, because the capillary vessels in the sub-plural connective tissue are obliterated, This may be the result of shrivelling and disappearance of the connective tissue newly formed from the exudation, as then, in consequence of the arrest of the metamorphosis of tissue between the blood and the exudation, endosmose and exosmose cannot duly taken place. It is not until after the lapse of months or years, when the fluid portion of the exudation has penetrated through the false membranes investing the pleura that its resorption occurs. In the first case, internal medicines can, of course, avail nothing, as in them we possess no means of exciting the reformation of vessels. What has been said explains the action of iodine injected in exudations, inasmuch as, by exciting inflammation, it causes the development of new vessels, and so induces the resorption of the effusion. But this view does not, perhaps, encourage us to the frequent employment of thoracentesis and subsequent injection of iodine; for this proceeding is by no means so safe as the corresponding operation for hydrocele. But, apart from that consideration, the injection of iodine or nitrate of silver into the pleural sac can answer no useful purpose; as, on the one hand, in consequence of the presence of the albuminous exudation, the caustic influence of these agents cannot reach the pleural sac, particularly as by the chemical combination which these substances form with the effusion, their power is altered and exhausted, so that the fluid must in the first instance be pumped but, which violent and sudden evacuation may be attended with evil consequences. But, on the other hand, in effusions of long standing, which have already attained to partial organization and shrivelling of the product of inflammation, the injection will be inefficacious, because the lung can now no longer fill the space previously occupied by the fluid exudation, espe cially as the investing false membranes must first be broken up by the lungs, which is not conceivable. But even if this should take place, a sudden evacuation could be followed by no favorable result, because necessarily there must be ruptures of the pleuritic adhesions and bursting of the compressed pulmonary parenchymal Therefore, in a pleuritic effusion of long standing, it is only exceptionally that puncture is admissible, when the exudation is so considerable as to depress the diaphragm, to displace the mediastinum, and so to compress the lung that danger of suffocation supervenes. But how can the resorption of pleuritic exudations be induced? Experience shows that all those means which lower the pressure of the blood, or augment the secretions, and therefore promote the separation of water from the blood, effect a diminution of the fluid effusion. Accordingly, venesections and diuretics may be indicated in cases of effusion; but these effects also occur spontaneously. In chronic exudations, which are already organized, such means will even be rather injurious, and the indication will be to employ remedies capable of dissolving solid exudations. Such remedies are iodine and mercury. The cautious employment of these means may therefore be adopted, and they are particularly suitable for external application. Professor Skoda has for some years employed these means experimentally, and has often seen pleuritic exudations rapidly diminish after the use of mercurial ointment, iodine ointments, iodide of glycerin, and black oxyde of copper in the form of ointment. It is self evident that in all chrnoic pleuritic effusions the diet must be good, in order as much as possible to counteract their injurious effects upon the system at large.—*Dublin Quart. Jour., Aug.* 1860.

